# The Endobiota Study: Comparison of Vaginal, Cervical and Gut Microbiota Between Women with Stage 3/4 Endometriosis and Healthy Controls

**DOI:** 10.1038/s41598-019-39700-6

**Published:** 2019-02-18

**Authors:** Baris Ata, Sule Yildiz, Engin Turkgeldi, Vicente Pérez Brocal, Ener Cagri Dinleyici, Andrés Moya, Bulent Urman

**Affiliations:** 1 Department of Obstetrics and Gynecology, Koc University Faculty of Medicine, Istanbul, Turkey; 2 Department of Obstetrics and Gynecology, Koc University Hospital, Istanbul, Turkey; 3 Área de Genómica y Salud, Fundación para el Fomento de la Investigación Sanitaria y Biomédica de la Comunidad Valenciana (FISABIO-Salud Pública), Valencia, Spain; 4 Eskisehir Osmangazi University Faculty of Medicine, Department of Pediatrics, Eskisehir, Turkey; 5 Institute for Integrative Systems Biology, Universitat de València, Valencia, Spain; 6 CIBER en Epidemiología y Salud Pública (CIBEResp), Madrid, Spain

## Abstract

Dysbiosis in the genital tract or gut microbiome can be associated with endometriosis. We sampled vaginal, cervical and gut microbiota from 14 women with histology proven stage 3/4 endometriosis and 14 healthy controls. The V3 and V4 regions of the 16S rRNA gene were amplified following the 16S Metagenomic Sequencing Library Preparation. Despite overall similar vaginal, cervical and intestinal microbiota composition between stage 3/4 endometriosis group and controls, we observed differences at genus level. The complete absence of Atopobium in the vaginal and cervical microbiota of the stage 3/4 endometriosis group was noteworthy. In the cervical microbiota, *Gardnerella*, *Streptococcus*, *Escherichia*, *Shigella*, and *Ureoplasma, all of which contain potentially pathogenic species*, were increased in stage 3/4 endometriosis. More women in the stage 3/4 endometriosis group had *Shigella/Escherichia* dominant stool microbiome. Further studies can clarify whether the association is causal, and whether dysbiosis leads to endometriosis or endometriosis leads to dysbiosis.

## Introduction

Endometriosis is a chronic estrogen dependent condition defined by the presence of endometriotic gland and stroma outside of the uterine cavity. It is commonly believed to affect approximately 10% of women, however recent studies suggest that the prevalence can be lower^1–7^. Ectopic endometriotic foci are most commonly present in the pelvis, but they are also found in other locations including but not limited to the intestine, diaphragm, and the pleural cavity^8,9^. Inflammation in the ectopic foci characterizes the disease^6^. While endometriosis is associated with dysmenorrhea, pelvic pain, dyspareunia and/or infertility, some women with the disease are asymptomatic^10^.

Seeding of viable endometrial cells to the pelvis by retrograde menstruation is the most plausible theory explaining pathogenesis of the condition^11^. However, occurrence of endometriosis in only 1–2% of women despite all women having retrograde menstruation to some extent suggests other mechanisms playing a role^12^. Epidemiologic data suggest a genetic predisposition, but many studies conducted to date failed to identify a single gene or a gene sequence^13,14^. Immunologic perturbations independently or in association with epigenetic changes could play a role in the development of the disease^15^.

All microorganisms, i.e. bacteria, archaea, protists, fungi and viruses, living in and on our body comprise the human microbiota^16^. The collective genomes of the microorganisms are named the microbiome. The human microbiome involves almost 150 fold more genes than the human genome itself^16^. Microbiota is important for our bodily functions. A well-known example is the need for gut microbiome for the synthesis of vitamins B12 and K, provision of intestinal mucosal integrity, prevention of invasion of harmful bacteria, and maturation of the immune system^17^.

Recent studies present strong evidence for an association between dysbiosis, i.e. disruption of the gut microbiota, and inflammatory bowel disease, neuropsychiatric diseases, psoriasis, arthritis, and some cancers, especially colon cancer^18–27^. This is explained by the potential immunoregulatory function of the gut microbiota playing a role in systemic inflammatory cellular responses. Since abnormal inflammatory response and activation of the immune cells in the peritoneal cavity are thought to play a role in the pathogenesis of endometriosis, an association between microbiota and endometriosis is likely^28^. Prior research has shown that dysbiosis leads to increased estrogen levels in the circulation^29^. Increased estrogen exposure can stimulate growth of ectopic endometriotic foci and inflammatory activity in them^6,30^. Thus, it may also be possible that the gut microbiota with their role in the regulation of the estrogen cycle, can be associated with endometriosis.

It is plausible that microbiota can play a role in development of endometriosis by affecting the host’s epigenetic, immunologic and/or biochemical functions. The present study aimed to compare gut, vaginal and cervical microbiota between women with and without endometriosis.

## Material and Methods

The study was conducted between 2016 and 2017 at the Gynecology and Endometriosis Clinics of Koç University Hospital in Istanbul, Turkey. The protocol of this prospective observational cohort study was approved by the Koç University Clinical Research Ethics Committee (2016.220.IRB1.027). All study related procedures were performed in accordance with relevant guidelines and regulations. All participants provided written signed informed consent.

### Participants

Reproductive aged women who had a histologic diagnosis of endometriosis comprised the study group, whereas asymptomatic reproductive aged women who presented for a routine well woman visit or preconceptional counselling were included in the control group.

Exclusion criteria were:

- <18 or >45 years of age on the day of sample collection.
- Being ever pregnant including the day of sample collection.
- Postmenopausal status.
- Having clinical signs and symptoms suggestive of endometriosis, i.e. dysmenorrhea, dyspareunia, dyschesia and/or infertility precluded recruitment to the control group. These symptoms were assessed with the Biberoglu-Behrmann (B&B) scale and women with a score >0 were not included in the control group^31^.
- Having taken antibiotics or probiotics within the last 8 weeks.
- Inflammatory bowel disease, functional bowel disease, history of gastrointestinal cancer or surgery, acute or severe gastrointestinal symptoms that require medical treatment, gastrointestinal infection or morbidity.
- An abnormal pap smear result within the last three years.
- Body mass index >30 kg/m2.

In addition, women who failed to achieve a pregnancy despite 12 months of regular unprotected intercourse were considered infertile and were not recruited to the control group.

### Sample collection

All participants provided stool samples at the clinic. Endometriosis patients, who would undergo surgery, provided the samples on the evening before surgery.

A minimum of 5 mL fresh stool sample was collected in a 15 mL Falcon tube and rapidly transferred to −80 °C to be stored in an upright position until DNA extraction.

Vaginal and endocervical swabs were collected following the insertion of a sterile vaginal speculum by one of the two gynecologists (SY, ET) with eNAT^TM^ kits (606CS01L, Copan Group, Copan Italia). Two separate swabs and collection tubes were used for vaginal and cervical samples. The swabs used to collect endocervical samples were not touched to the vaginal walls during sample collection. Both samples were immediately transferred to −80 °C to be stored in an upright position until DNA extraction.

### DNA Extraction

Following the completion of sample collection, they were all transferred on dry ice to Diagen laboratory located in, Ankara, Turkey for DNA extraction. Fecal samples were weighed to extract total DNA using the QIAamp DNA Stool Mini Kit (Qiagen**®**, Hilden, Germany), in accordance with the manufacturer’s instructions. Kurabo QuickGene DNA tissue kit S (DT-S) (Japan) was used for cervical and vaginal samples, according to the manufacturer’s instructions.

### Microbiome analysis

Extracted DNA samples were shipped on dry ice to FISABIO, Valencia, Spain for further analysis.

### 16S rRNA gene amplification, library construction, and sequencing

The V3 and V4 regions of the 16S rRNA gene were amplified following the 16S Metagenomic Sequencing Library Preparation Illumina protocol (Part # 15044223 Rev. A, Illumina, CA, USA). Extraction controls were amplified and sequenced in parallel with the samples.

The primers targeting this region used were 16S Amplicon PCR Forward Primer = 5′-TCGTCGGCAGCGTCAGATGTGTATAAGAGACAGCCTACGGGNGGCWGCAG-3′ and 16S Amplicon PCR Reverse Primer = 5′-GTCTCGTGGGCTCGGAGATGTGTATAAGAGACAGGACTACHVGGGTATCTAATCC-3′.

A total of 12.5 ng of genomic DNA per sample was used for amplification under the following PCR conditions: 5 min of initial denaturation at 94 °C followed by 25 cycles of denaturation (30 s at 94 °C), annealing (30 s at 52 °C) and elongation (1 min at 72 °C). After amplification, the products were visualized in 1.4% agarose gels and quantified using a Qubit^®^ 3.0 Fluorometer (Thermo Fisher Scientific, Carlsbad, CA, USA). Next, the multiplexing step was performed with Nextera XT Index Kit (Illumina) by attaching dual indices to both ends of the PCR products. The samples were pooled in equimolar amounts and sequenced in the Sequencing Service facilities of FISABIO using a 2 × 300 bp paired-end run using the MiSeq® Reagent kit v3, (Illumina), on a MiSeq Sequencer according to manufacturer’s instructions (Illumina).

We used internal controls during extraction, PCR (there is a negative control for PCR) and sequencing. During sequencing, we used internal control for 16S and it contained 1007 reads.

### Sequence Bioinformatics analysis

Primary processing of sequencing reads was carried out on the raw reads starting by a quality assessment performed by the use of prinseq-lite program applying the following parameters: min_length: 50, trim_qual_right: 30, trim_qual_type: mean and trim_qual_window: 20^32^. We used Prinseq-lite (v0.20.4) (webpage: http://prinseq.sourceforge.net). Forward and reverse reads passing the quality check were joined using FLASH program applying default parameters^33^. Next, joined, unjoined reads and singletons were concatenated and mapped against the human genome (GRCh38.p11, reference human genome, Dec 2013) by using Bowtie2^34^ with end-to-end and very sensitive options (–very-sensitive: -D 20 -R 3 -N 0 -L 20 -i S,1,0.50).

Human-unaligned files were taxonomically analyzed by aligning the reads to the Ribosomal Database Project (RDP) database, using the naïve Bayesian rdp_classifier 2.12 tool that provides taxonomic assignments from domain to genus^35^. The resulting files were parsed to get the counts for each taxon in each sample, and finally a unique contingency table was generated. The contingency table was converted into Biom format, using the QIIME pipeline version 1.9.0 for composition and abundance analyses, as well as for ecological diversity^36^. For diversity within samples, or alpha diversity, 1,000 rarefactions of 9,500 random reads per sample, with replacement, were carried out and the alpha diversity was calculated with Shannon diversity index (SI). Boxplots were created using the free statistical package R 3.1.0^37^. Diversity between samples, or beta diversity, was assessed with Principal Coordinates Analysis (PCoA) using Bray-Curtis dissimilarity index matrices, implemented by QIIME pipeline, to create linear combinations (multidimensional scaling) that explain the data better. Statistical significance of sample groupings was calculated by using the resulting distance matrices and the Adonis nonparametric analysis of variance^38^. Additional analyses, such as non-parametric unpaired two-samples Wilcoxon tests for groups, sample types, and both, were carried out with R scripts. Our nucleotide sequence data for 16S rRNA gene was deposited in EBI Short Read Archive (https://www.ebi.ac.uk/ena) under the study accession number PRJEB26800 with accession numbers ERS2487953 to ERS2488036.

## Results

### Participant characteristics

Fourteen Caucasian women with endometriosis and 14 Caucasian healthy controls were included in the study. Median (25^th^–75^th^ percentile) age, (28.5 (26–31.3) vs 27.5 (25.8–30) years; p = 0.54) and median (25^th^–75^th^ percentile) body mass index, (23 (21–24.3) vs 21 (20.1–24.2) kg/m^2^; p = 0.25), were similar in the endometriosis and control groups, respectively. None of the participants were on oral contraceptives or had an intrauterine device. None reported vaginal intercourse during the three days before sample collection. Similar numbers of women in each group provided the samples during the follicular (7/14 in both groups) or luteal phase (7/14 in both groups) of the menstrual cycle (p>0.99).

All women in the endometriosis group had moderate to severe (stage 3–4) endometriosis according to American Fertility Society Revised Classification^39^. All patients had endometriomas and widespread lesions in the pelvis, all had deep infiltrating endometriotic nodules on one or both of the sacrouterine ligaments, and partially or completely obliterated pouch of Douglas. While all women had superficial bowel involvement effecting serosal surface only, two of the 14 women were considered to have bowel involvement affecting muscular and/or mucosal layer of the sigmoid colon.

Cervical, vaginal and stool samples were obtained from each participant. In a set of 84 samples, we identified 327 different bacterial genera, a total of 6,160,862 reads, with an average of 73,344 ± 24,482 reads per sample (ranging from 142 to 110,467). Our cut-point analyses were 9500 reads, and that excluded one sample (XS1_3H5 142 reads), while having a high number of reads for consistent analysis.

### Diversity Analyses

**Figure 1:**
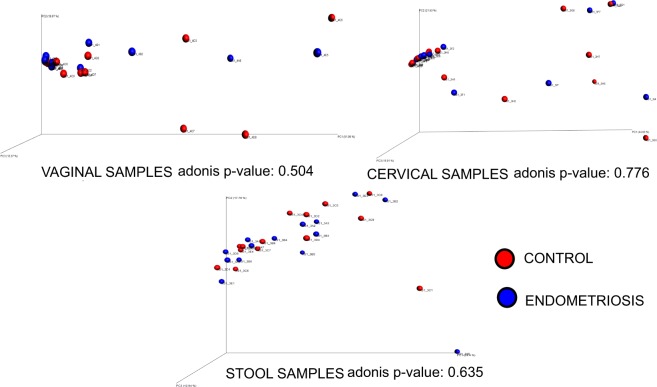
Principal Coordinates analysis showing the distribution of the vaginal, cervical and stool samples, based on Bray-Curtis dissimilarity matrices. Blue dots indicate control group (n = 14), red dots, stage 3–4 endometriosis group (n = 14).

**Figure 2:**
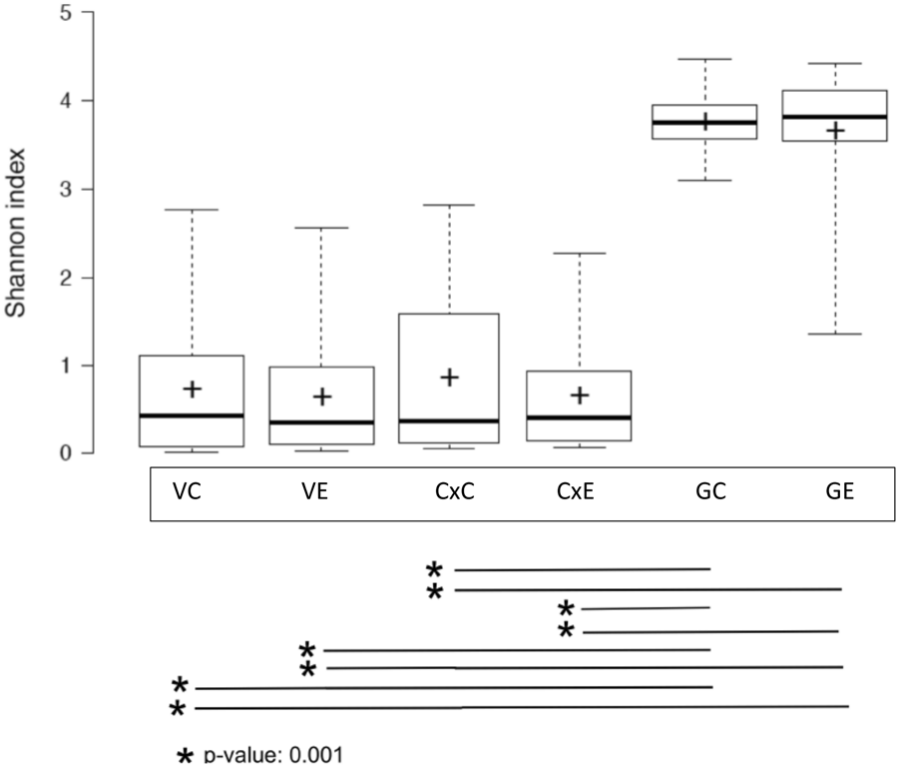
Boxplots depicting the Shannon diversity index of the cervical (Cx), gut (G) and vaginal (V) microbiota in control (C) (n = 14) and endometriosis (E) (n = 14) groups. Boxes indicate the first and third quartiles, dash lines the upper and lower whiskers, crosses indicate the mean, and horizontal bold lines the median.

Overall, PCoA analysis shows that vaginal, cervical and gut microbiota composition was similar between the endometriosis group and controls (Fig. 1). Non-parametric adonis tests showed no significant differences according to the condition status in all niches (Fig. 1). Bacterial diversity measured by SI was similar for vaginal, cervical, and gut samples between from the endometriosis and the control groups (Fig. 2). The fecal samples had higher diversity than both the cervical and vaginal samples (Fig. 2).

### Genus Analysis

**Figure 3:**
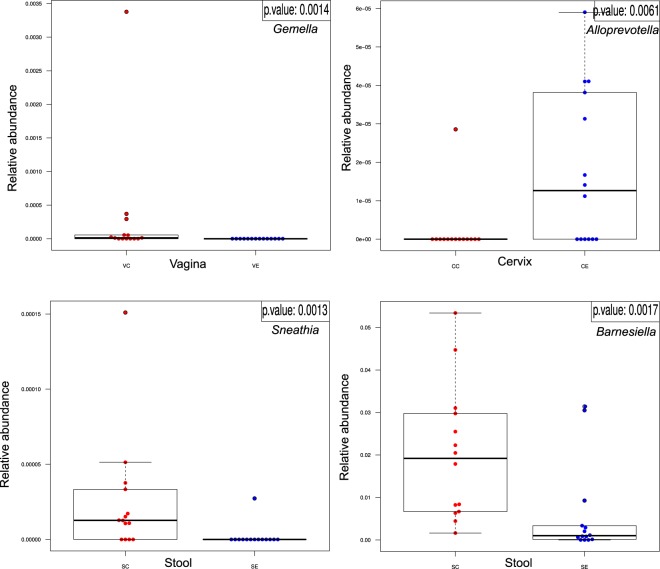
Sample graphs showing some of the genera which had different abundance between stage 3–4 endometriosis (n = 14) and controls (n = 14).

**Figure 4:**
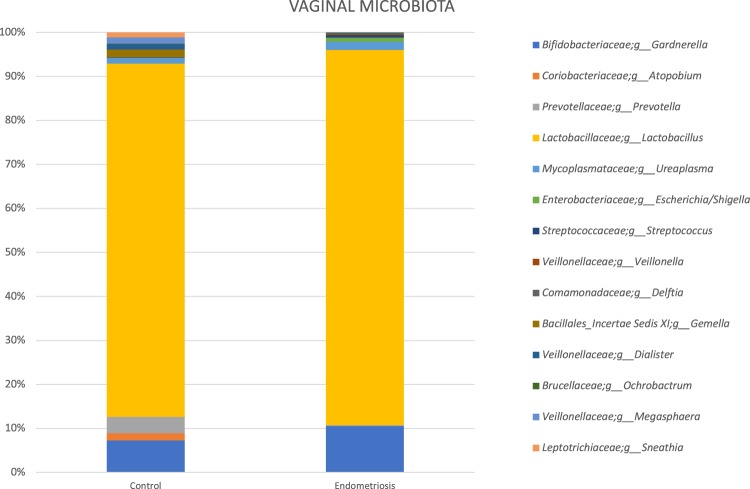
Most abundant taxa (at genus level) among healthy controls (n = 14) and women with stage 3–4 endometriosis (n = 14) in vaginal samples.

**Figure 5:**
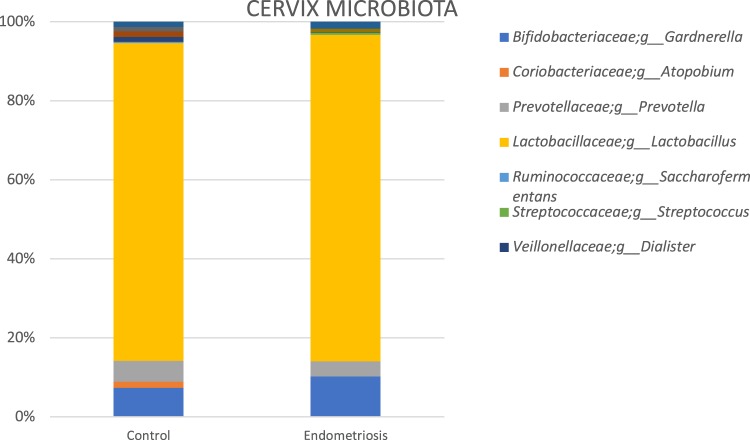
Most abundant taxa (at genus level) among healthy controls (n = 14) and women with stage 3–4 endometriosis (n = 14) in cervical samples.

**Figure 6:**
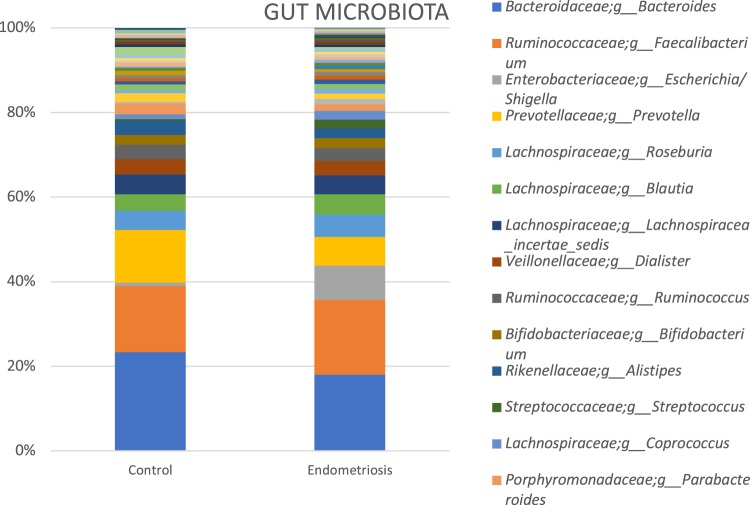
Most abundant taxa (at genus level) among healthy controls (n = 14) and women with stage 3–4 endometriosis (n = 14) in stool samples.

We performed Wilcoxon tests for the comparison of pairs of groups and only for bacteria, which were present in at least half of the samples of at least one of the two groups. For vaginal samples, at genus level, the complete absence of Gemella and *Atopobium* in the endometriosis group was noteworthy. In cervical samples, at genus level, Atopobium and *Sneathia* were completely absent, while *Alloprevotella* was significantly increased in the endometriosis group (p < 0.01 for both). For stool samples, genera *Sneathia*, *Barnesella* and *Gardnerella* were significantly decreased in the endometriosis group (p < 0.01 for all). (Figs 3–6 and Table 1)

**Table 1:** Differences between microbiota in women with endometriosis and healthy controls.

Niche	Decreased	Increased
Vagina	Gemella* Atopobium*	—
Vagina excluding Lactobacillus	—	Gardnerella Escherichia/Shigella
Cervix	Atopobium* Snethia	Alloprevotella
Cervix excluding Lactobacillus	Prevotella Dialister Megasphaera	Gardnerella Streptococcus Escherichia/Shigella Ureaplasma
Stool	Gardnerella Snethia Barnesella	

*Completely absent.

There were 25 different observed genera, the majority being *Lactobacillus* (80–84%), in the cervical microbiota of the endometriosis group and controls. In patients with endometriosis, 84.6% were *Lactobacillus* and 10.5% *Gardnerella*, making 95.1% of the total bacteria. In the control group, *Lactobacillus* comprised 80.2% and *Gardnerella* 7.3% of total bacteria.

**Figure 7:**
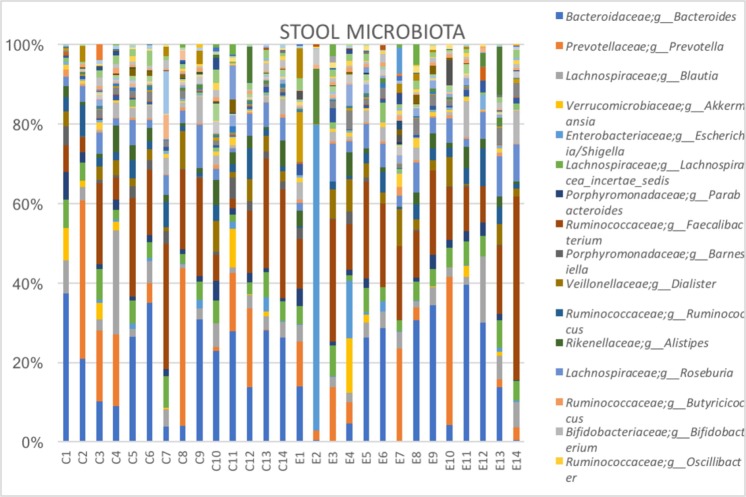
Bacterial abundance in stool samples from controls (n = 14) and stage 3–4 endometriosis (n = 14) group. Increased Escherichia/Shigella abundance is observed in E2 and E4 who later required segmental colon resection due to bowel involvement.

Two patients in the endometriosis group, had more *Escherichia/Shigella* in stool, and further follow-up of these patients showed severe bowel involvement by endometriosis requiring segmental colon resection (Fig. 7).

### Sensitivity Analyses Excluding Lactobacillus

Sensitivity analyses of vaginal microbiota after exclusion of *Lactobacillus* species, showed that *Gardnerella* comprised a significantly higher proportion of the remaining microbiota in endometriosis group than in controls (72.9% vs 36.8%, p < 0.05). *Escherichia*/*Shigella* was also more abundant in the endometriosis group; *Prevotella* and *Dialister* were decreased in patients with endometriosis compared to the controls, without statistical difference.

Sensitivity analysis excluding *Lactobacillus* species showed that *Gardnerella* comprised a significantly higher proportion of the remaining microbiota in the cervix of endometriosis group than in controls (67.7% vs 36.8%, p < 0.05). *Streptococcus*, *Escherichia*/*Shigella* and *Ureaplasma* were also more abundant in the endometriosis group. However, *Prevotella*, *Dialister*, and *Megasphaera* were significantly decreased in patients with endometriosis compared to the controls. The complete absence of particular genus, *Atopobium* in the endometriosis group was noteworthy (Figs 4 and 5).

## Discussion

Overall, vaginal, cervical and gut microbiota composition was similar between women with stage 3–4 endometriosis and controls. However, there were some differences between bacteria groups. Noteworthy are, the absence of a particular genus, *Atopobium in vaginal and cervical microbiota*, increased *Gardnerella* presence in cervical microbiota, and more women having *Escherichia* and *Shigella* dominant gut microbiota in the endometriosis group.

Even though SI is a good tool to assess microbiota diversity in anatomical sites where there are many genera, e.g. stool, it can have limitations in sites where there are only a few genera like the vagina and the cervix^40^. In our sample, there were 230 genera in the stool and 182, 183 in the vagina and cervix, respectively. Thus, the observed differences in some bacteria could be still relevant. In addition, the lower genital tract of reproductive aged females is unique as it is predominantly populated by a single genus, i.e. *Lactobacillus*, which is also the most abundant genus in endometrial microbiota, a fact that could limit the value of SI as an assessment tool^41–43^. Therefore, we undertook sensitivity analyses by excluding Lactobacillus, and the following bacteria were found to be significantly increased; *Sneathia, Gardnerella, Streptococcus*, *Escherichia*/*Shigella* and *Ureaplasma*, while *Alloprevotella* was significantly decreased in the cervix.

If confirmed in other studies, the complete absence of *Atopobium* in the vagina and cervix, together with the increased presence of *Gardnerella, Escherichia/Shigella* and *Ureoplasma* in cervical microbiome of patients with endometriosis could be a relevant finding of this study. Atopobium is recently implicated as a gynecological pathogen associated with bacterial vaginosis, obstetric bacteremia, and, possibly, with endometrial cancer^44–47^. Intriguingly, a recent study comparing uterine microbiome between women with endometrial cancer and with benign pathologies, reported the presence of Atopobium *vaginae* in 14/15 women with endometrial cancer, as opposed to 4/10 women with benign pathologies^46^. The authors suggest that Atopobium can facilitate infection by Porphyromonas species, which can be present intracellularly and disrupt cell regulatory functions eventually leading to a carcinogenic trigger^46^. Whether the association is causal is unclear, but from a different perspective, maybe the absence of Atopobium can be related to occurrence of endometriosis, which is also a benign gynecologic pathology, through a different downstream effect.

If there is indeed an association between endometriosis and female microbiota composition, what would be the direction of causation; altered immune response leading to both endometriosis and different microbiota composition or different microbiota, including the mere presence or absence of a single species, leading to altered immune function and the disease? While microbiome could be a useful screening/diagnostic tool for endometriosis in both scenarios, it could become a therapeutic target in the latter. Indeed, a former study including 95 women who underwent surgery for benign gynecologic conditions, not known to be related to infectious etiology, reported subtle differences between uterovaginal microbiomes of patients with adenomyosis, infertility due to endometriosis, and fibroids^41^. However, the study cohort lacked healthy controls and comparators for endometriosis associated infertility included fertile women whose microbiome could have changed due to past pregnancy^48,49^. Our study is unique in the sense that microbiome between nulligravid healthy controls and endometriosis patients were compared.

It is intriguing that two women in the endometriosis group had *Escherichia/Shigella* dominant gut microbiome, while none of the controls showed a similar composition. It is even more interesting that these two women underwent segmental colon resection as part of surgical treatment of deep infiltrating endometriosis during the period between sample collection and the microbiome analyses, i.e. the surgeons were not aware of microbiome composition. It is not always possible to assess the depth of bowel involvement preoperatively with imaging, and if confirmed, gut microbiome analysis could be an additional tool to predict the possibility of bowel resection and to counsel the patients.

The advantages of this study are strict selection criteria reflected by stability of microbiota, exclusion of ever pregnant women, and women with other conditions/medications that could affect the microbiome. All participants belonged to the same ethnicity. Whether vaginal and/or cervical microbiota varies across the menstrual cycle is controversial^41,50^. Yet, similar numbers of women provided samples during the follicular and luteal phases in both groups, and observed differences and similarities between the two groups are unlikely to be altered by menstrual cycle. Importantly, inclusion of women with histology proven endometriosis is an advantage. The lack of laparoscopic confirmation of the absence of endometriosis in the control group could be regarded as a shortcoming of our study. However, women in the control group were asymptomatic and did not have any ultrasound findings of the condition with B&B scores of zero, rendering them very unlikely to have endometriosis. Moreover, even if some women in the control group had mild endometriosis that could not be diagnosed with history and pelvic examination, this would have made the study groups more similar leading to underestimation of any differences between them. Thus, the observed differences in the present study are unlikely to be overestimates. The absence of whole genome and metagenomic analyses and endometrial samples are other limitations, however, collection of endometrial samples without contaminating with cervical microbiome is not feasible.

In conclusion, while overall microbiome composition in the cervix, vagina and gut seems similar between women with stage 3–4 endometriosis and healthy controls, there seems to be some differences at the genus level. Further studies are needed to analyze an association between endometriosis and microbiota.
